# A Retrospective Multicentric Study of Ewing Sarcoma Family of Tumors in Patients Older Than 50: Management and Outcome

**DOI:** 10.1038/s41598-017-17733-z

**Published:** 2017-12-20

**Authors:** Pauline Rochefort, Antoine Italiano, Valérie Laurence, Nicolas Penel, Audrey Lardy-Cleaud, Olivier Mir, Christine Chevreau, Francois Bertucci, Emmanuelle Bompas, Loic Chaigneau, Dominique Levy, Thomas Ryckewaert, Sarah Dumont, Pierre Meeus, Dominique Ranchere, Jean-Yves Blay, Philippe Alexandre Cassier

**Affiliations:** 1 Department of Medical Oncology, Centre Léon Bérard, Lyon, France; 2 Department of Medical Oncology, Institut Bergonié, Bordeaux, France; 3 Department of Medical Oncology, Institut Curie, Paris, France; 4 Department of Medical Oncology, Centre Oscar Lambret, Lille, France; 5 Department of Medical Oncology, Gustave Roussy, Villejuif, France; 6 Department of Medical Oncology, Institut Claudius Regaud, Toulouse, France; 7 Department of Medical Oncology, Institut Paoli Calmettes, Marseille, France; 8 Department of Medical Oncology, Centre René Gauducheau, St. Herblain, Saint-Herblain, France; 9 Department of Medical Oncology, Hôpital Jean Minjoz, Besançon, France; 10 Department of Pathology, Centre Léon Bérard, Lyon, France; 11 Université Claude Bernard, Lyon, France

## Abstract

Ewing’s sarcoma family of tumors (EFTs) is a group of rare and aggressive tumors. Data on EFTs in patients (pts) ≥ 50 years are limited and these pts are often not eligible for clinical trials. Some, but not all, studies have reported inferior outcome for older pts with EFTs. We conducted an IRB-approved retrospective analysis among centers of the French Sarcoma Group on pts diagnosed with EFTs at age ≥50 between 2000 and 2012. Clinical features, treatment modality and outcomes were analyzed. Seventy-seven pts were identified, including 36 females (46.8%) and the median age at diagnosis was 56 years (range: 50–86). The primary tumor was located in soft tissue in 59 pts (76.6%). Fifty-six pts (72.7%) had localized disease, among them 49 (87.5%) received chemotherapy in addition to local therapy. Their estimated 3-yr OS and event-free survival (EFS) rates were respectively 73.3% and 62.2%. Recurrence occurred in 43 pts. The estimated 3-yr OS rate was 37% in pts with metastatic disease at presentation. EFTs in pts ≥50 years are more likely to originate from soft tissue and their outcomes appear to be worse than that of younger pts treated with modern protocols.

## Introduction

The Ewing’s sarcoma family of tumor (EFTs) is a group of aggressive tumors affecting most often children, adolescent and young adult^1^,with a median age of 15 years at diagnosis. The estimated annual incidence of Ewing sarcoma is 3/1.000.000 in France and in the USA^2,3^. In 85–90% of EFTs cases, the tumor is characterized by a translocation involving the EWSR1 (EWS RNA-Binding Protein 1) gene on chromosome 22, and an ETS (E26 transformation-specific)-family gene such as FLI-1 or ERG^1^. The primary site of disease is the bone in 80% of cases and about one fourth of patients present with metastasis at initial diagnosis^1^.

The current standard of care for patients with EFTs includes intensive induction chemotherapy followed by local therapy (surgery, radiotherapy or a combination of both) followed by adjuvant chemotherapy^4^. Recent studies have shown the benefit of four or five drug regimens and interval time compression of chemotherapy, suggesting a beneficial effect of dose-intensity in this chemo-sensitive disease^5–7^. However, most of these studies have shown that the benefit of more intensive chemotherapy regimens is limited to patients with localized disease^8^.

Although rare, EFTs in older subjects represents a clinical challenge because many of these patients will not be suitable candidates for intensive induction chemotherapy. Therefore, the optimal management of these patients remains to be defined. Limited data are available on age-specific prognostic and treatment, with discordant conclusions. A recent report for the SEER database has shown an inferior survival for EFTs patients older than 40 years^9^, while three other reports suggest that adherence to chemotherapy protocol is similar in patients less than 40 and patients older than 40^10–12^. Authors of these reports therefore argue that older patients should be managed with similar chemotherapy protocols. These studies have in most cases focused on patient treated in the 1990’s, before the advent of 5 drug regimens. Furthermore, some have compared the outcome of patients less than 40 to that of patients aged 40 to 50 years^11,12^.

The EURO EWING 99 study has served as the guide for the management of patients with EFTs in the French Sarcoma Group (FSG) from 2000 to 2012, however only patients 50 years or younger were eligible. The objective of the present study was to describe the management and outcome of older patients and compare them to that of younger patients^10^.

## Results

### Patients and tumors’ characteristics

Seventy-seven patients were enrolled, including 36 females (46.8%) and their characteristics are described in Table 1. Briefly, the median age at diagnosis was 56 years (range: 50–86) and performance status was ≤1 for 57 patients (74%). The primary tumor was located in soft tissue in 59 pts (76.6%) and in bone in 18 (23%). Sites of extra-osseous tumors included: gluteal muscle, retroperitoneum, rhinopharynx, pleura, cervical muscles. Median tumor size was 6.8 cm and tumor size was >8 cm in 25 patients (40%). Fifty-six patients (73%) had localized disease whereas 21 (27%) presented with metastases at diagnosis and among them, 11(52%) had lung-only metastases. There were no statistical differences between stage at diagnosis for patients <65 and ≥65 years (p = 0.84).

**Table 1:** Patients characteristics.

**Characteristics**	**All (n = 77)**		**Localized (n = 56)**	
	**N**	**%**	**N**	**%**
Age				
<65 years	66	85.7%	47	83.9%
≥65 years	11	14.3%	9	16.1%
Gender				
Male	41	53.2%	32	57.3%
Female	36	46.8%	24	42.8%
Primary tumor				
Soft tissue	59	76.6%	45	80.4%
Bone	18	23.4%	11	19.6%
Tumor location				
Abdomen	10	13%	6	10.7%
Head and neck	7	9.0%	6	10.7%
Lower extremity	21	27.3%	17	30.3%
Upper extremity	6	8.0%	6	10.7%
Pelvis	5	6.5%	3	5.4%
Spinal column	14	18.1%	9	16.1%
Thorax	14	18.1%	9	16.1%
Size				
<8 cm	37	48.0%	32	57.1%
≥8 cm	25	32.5%	17	30.4%
Missing data	15	19.5%	7	12.5%
Stage				
Localized	56	72.7%		
Metastatic	21	27.3%		
Lung only (n = 10)				
Bone (n = 8)				
Multiple sites without bones (n = 3)				
Translocation				
EWSR1-FLI1	26	33.7%	21	37.5%
EWSR1-ERG	1	1.3%	1	1.8%
EWSR1-NOS	22	28.6%	14	25.0%
None	4	5.2%	2	3.6%
Missing data	24	31.2%	18	32.1%

NOS: not otherwise specified.

Molecular analysis was available for 53 patients (69%): EWSR1-FLI1 fusion was found in 26 patients, FUS-ERG in one patient and rearrangement of the EWSR1 gene not otherwise specified (NOS) in 22 patients (Table 1).

### Treatment modality

Thirty-seven patients (48%) were initially managed outside of a FSG institution. Their survival was not statistically different from those initially treated in FSG group. Six of these patients were managed with surgery alone and one with radiotherapy alone.

Among 56 patients with localized disease, 49 (88%) received chemotherapy in addition to local therapy and among these, chemotherapy was considered intensive in 34 patients (61%) (Table 2). Most of the patients who received intensive chemotherapy were younger than 65 years (p = 0.025). The median number of cycles given was six (range 0–17). Median cumulative administrated dose was 341 mg/m^2^ for doxorubicin and 49.000 mg/m^2^ for ifosfamide. Timing of chemotherapy (neoadjuvant versus adjuvant) was not associated with significant survival difference. Local therapy was surgery in 20 patients, surgery and radiotherapy in 28 patients and radiotherapy alone in five patients. Median radiation dose was 50.3 Gy (range 12–66). Twenty-nine patients (55%) with localized disease were treated with surgery first.

**Table 2:** Characteristics of patients, with localized disease. The total number of patients with localized disease was 56 but one patient who received chemotherapy was excluded from analysis because of missing data for chemotherapy dose.

**Characteristics**	**Intensive (n = 34)**		**Non intensive (n = 14)**		**No chemotherapy (n = 7)**	
	**N**	**%**	**N**	**%**	**N**	**%**
Age						
<65 years	31	91%	9	60%	6	86%
≥65 years	3	9%	5	40%	1	14%
Gender						
Male	21	62%	9	64%	1	14%
Female	13	38%	5	36%	6	86%
Primary tumor						
Soft tissue	29	85%	9	64%	6	86%
Bone	5	15%	5	36%	1	14%
Size						
<8 cm	21	62%	7	50%	4	57%
≥8 cm	10	29%	5	36%	2	29%
Missing data	3	9%	2	14%	1	14%
Translocation						
EWSR1-FLI1	16	47%	3	21%	2	29%
EWSR1-ERG	1	3%	0	0%	0	0%
EWSR1-NOS	8	24%	3	21%	3	43%
None	2	6%	0	0%	0	0%
Missing data	7	20%	8	58%	2	29%

NOS: not otherwise specified.

All patients with metastatic disease (n = 21) at presentation received chemotherapy, except three who had bulky primary tumors. One patient had surgery, while another had local radiotherapy but both died rapidly after local treatment. A third patient died of myocardial infarction three weeks after diagnosis, without receiving any specific treatment. Among patients receiving chemotherapy for metastatic disease, chemotherapy was considered intensive for 11 patients. Eight patients (38%) with metastatic disease had surgery of their primary tumors.

### Survival

**Figure 1:**
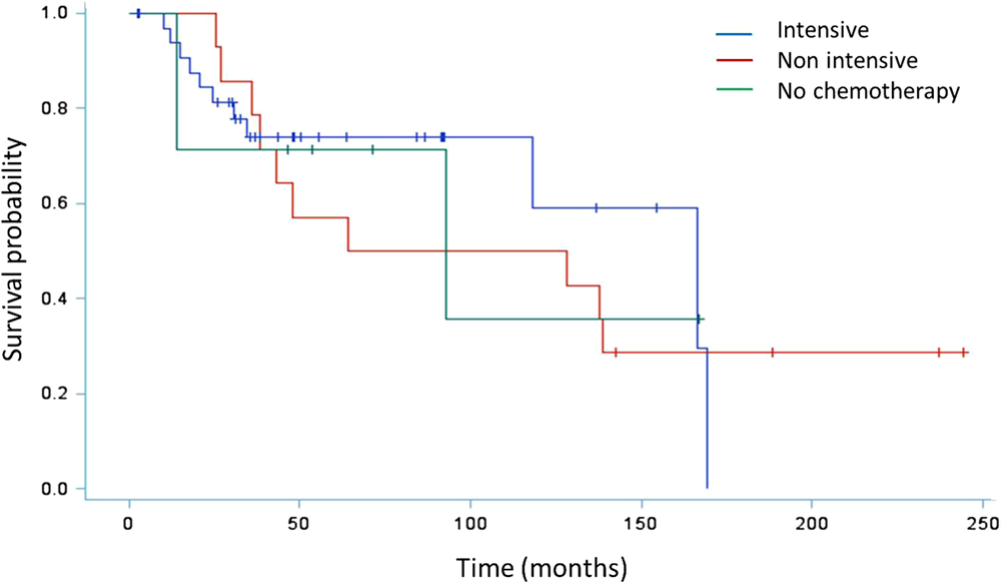
Event free survival of patients with localized disease according to chemotherapy regimen (intensive/non intensive/no). Kaplan Meier plots for event free survival, calculated from date of diagnosis to first event (progression, death) or date of last patient contact or date of death (of any cause). Log Rank: 0.8168 Chemotherapy regimens which contained high doses of anthracycline and high dose alkylating agents were considered intensive.

Median follow-up was 71 months and median overall survival (OS) was 92.8 months for the whole cohort, 128 months for patients with localized disease and 23 months for patients with metastatic disease. There was no significant survival difference between centers. For patients with localized disease, the estimated 3-year OS and event free survival (EFS) rates were respectively 73.3% (95% CI: 59.1; 83.3) and 62.2% (95% CI: 47.7; 73.8). There was no difference in OS or EFS between patients receiving intensive chemotherapy vs standard dose or no perioperative chemotherapy (Fig. 1). For patients with metastatic disease at presentation, the estimated 3-yr OS rate was 37% (95% CI: 15.7; 58.7).

In univariate analysis only initially high LDH level were marginally associated with worse overall survival in patients with localized disease. Other prognosis factor established in pediatric population such as age, tumor size, tumor location were tested but were not significantly associated with OS (Table 3).

**Table 3:** Univariate analysis of survival analysis for patients with localized disease.

**Variable**	**No. of patients**	**Hazard ratio**	**CI 95%**	***p***
Age				
<65 years	47	1.564	[0.706–3.466]	0.2704
≥65 years	9			
Tumor site				
Other	33	0.61	[0.260–1.431]	0.2557
Extremity (upper + lower)	23			
Size (cm)				
<8	32	1.621	[0.669–3.929]	0.2847
≥8	17			
LDH				
>Normal (N)	21	3.044	[1.010–9.174]	0.048
>N	9			
Chemotherapy*				
No	7			0.9301
Low-ose	14	1.041	[0.281–3.854]	
High-dose	34	0.88	[0.244–3.169]	
Anthracyclin				
No	8	0.683	[0.231–2.016]	0.49
Yes	47			
Vincristin				
No	23	0.916	[0.409–2.056]	0.8324
Yes	28			

*The total number of patients with localized disease who received chemotherapy was 49 but one patient who received chemotherapy was excluded from analysis because of missing data for chemotherapy dose.

CI 95%: confidence interval.

P: p value of Chi2 test.

### Management of recurrent disease

**Figure 2:**
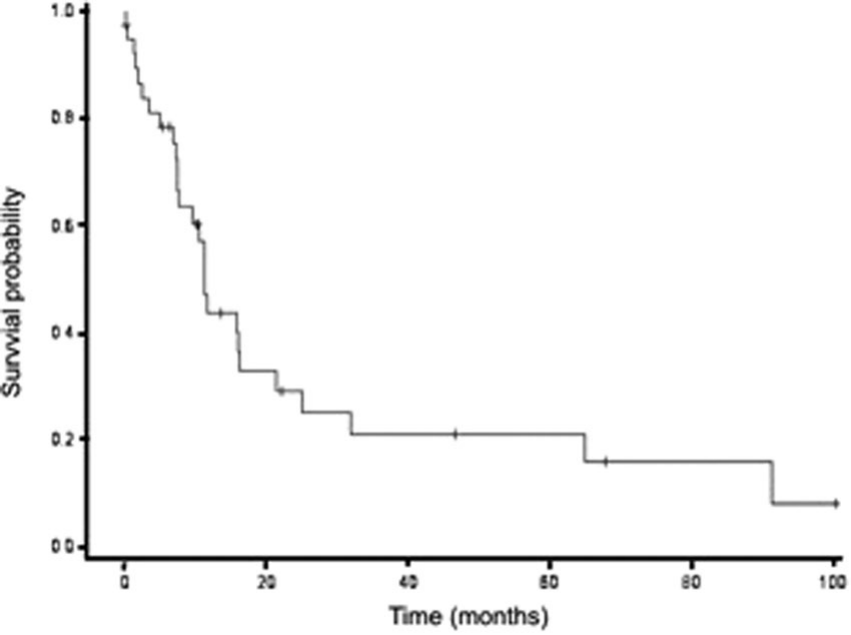
Post-recurrence survival. Kaplan Meier plots for post-recurrence survival in patients (localized + metastatic) who first achieved remission (n = 39), calculated from time of recurrence to death.

Forty-three patients (55%) experienced recurrence, survival data was available for 39 patients with a median time to recurrence of 18.2 months (range: 2–134). Recurrences were most often metastatic only (58.1%), followed by local only (23.2%). The site of recurrence was lung for 16 patients (37.2%) and bone for nine patients (20.9%). Median post-recurrence survival was 11.2 months (Fig. 2). Most patients (n = 32/43) received second line chemotherapy for recurrent disease with various chemotherapy regimens including cisplatine + etoposide; doxorubicin + cyclophosphamide; temozolomide + irinotecan and vinorelbine + cyclophosphamide.

## Discussion

There are few published studies on adult patients with EFTs and there results are conflicting. Some studies reported similar outcomes for adult and pediatric patients with EFTs. However, median age in these studies was relatively low, for instance: 21.5 years in Fizazi study^13^ and 27.1 years in Ahmed study^14^. Three studies reported similar outcomes for patients over 40 years old with EFTs^10–12^. Other studies reported shorter survival for adult patients^15–19^. Recent retrospective analyses of large cohorts of patients with EFTs have shown older age to be significantly associated with poorer prognosis^20–23^. A recent analysis from the SEER database has shown an inferior survival for EFTs patients older than 40 years^9^. One of the main questions to address is whether this difference in survival is due to differences in management or intrinsic differences in tumor and/or host biology. Recently published data in patients with synovial sarcoma suggests that there are biological differences between tumors in younger vs older patients^24^ but similar data are not available for EFTs.

We tried to address this issued in the present study, and we analyzed treatment modalities and outcome of older patients with EFTs. Several similarities in disease presentation with younger patients were noted such as a slight male predominance^25^, a predominance of non-bulky tumors (<8 cm) and localized disease at presentation (≈75%). The most common sites of metastasis and recurrence were the lung and the bone, which is consistent with EFTs in younger patients.

As previously reported, the majority of patients (77%) had soft tissue primary tumors, which is very different from EFTs in younger patients who present in the majority of cases with bone primaries^3,10–12^. Older series emphasized the need to treat extraosseous EFTs with EFTs protocol^26^, while recent studies suggest that extraosseous origin may be a favorable prognosis factor, at least in the pediatric population^27,28^. Our data suggest that the outcome of older patients with EFTs may be worse than in younger patients. Indeed, the 3-year OS rate in our study was 73.3% in patients with localized disease while the 3-year OS rate was 85.7% for patients with localized disease treated with VIDE as induction chemotherapy, and VAC or VAI as consolidation treatment in the EURO-EWING99 trial. Similarly, the Children Oncology Group reported a 5-year OS rate of 83% for patients with localized EFTs treated with compressed VDC-IE protocol and 77% for patients with standard interval^7^. These differences should, however, be interpreted with caution as our analysis is retrospective and included unselected patients as opposed to selected clinical trials patients described in the EURO-EWING99 and COG AEWS0031 studies. Nevertheless, we observed high recurrence rate (55%) in our cohort compared to pediatric series (recurrence rate 30%^29^), associated with a short post-relapse survival (median 9.5 months). The median time to recurrence of 18 months was however comparable to those reported in series of younger patients^29,30^.

For patients with metastatic disease at diagnosis, prognosis appears similar to that of the pediatric population with an estimated 3-year OS rate of 37%. However, outcomes for patients with metastatic tumors are heterogeneous, depending on metastatic sites (lung-only versus other)^31,32^, which therefore limits comparisons.

We observed significant differences with regards to treatment modalities: many patients in this study did not receive preoperative chemotherapy. In most cases, these patients were initially managed in non-expert centers. Atypical presentation (age, soft tissue involvement) may have led to misdiagnosis in first intention and consequently affect therapeutic strategy.

The worse prognosis of adult patients in previous studies has been attributed to less intensive treatment^9,17^. However, we did not observe any survival differences between patients receiving intensive chemotherapy versus non-intensive in our study. Interpretation of these results is however, limited by the small size of our study. Because many studies conducted in EFTs have overall shown an improvement in outcome with more intensive chemotherapy regimens^33–36^, we can only hypothesize that the inferior outcome may be related to treatment toxicity and difficulty to complete optimal treatment. We were not able to collect toxicity data in our retrospective study. The feasibility of intensive induction chemotherapy in older patients, with higher comorbidity remains uncertain. Subgroup analysis of the EE99 trial revealed no increase in toxicity in adult patients (19–50 years) who were not shown to have more dose modifications or delays than younger patients^37^. Other studies reported significantly higher toxicity and lower mean dose intensity of treatment for older patients compared to pediatric patients^10,17^.

Differences in clinical presentation and outcomes may support the hypothesis of biologic/genomic differences of EFTs occurring in adult than in children, as recently shown in synovial sarcoma^38^. EFTs are characterized by pathognomonic EWSR1 gene translocation with a member of the ETS transcription factor family and genomic studies have shown that the number of additional mutations or genomic alterations increased with age^39,40^, which suggests increasing genomic instability with age. The clinical relevance of these additional mutations is uncertain.

Clinical presentation and outcomes of patients diagnosed with EFTs at age over 50 years are different from pediatric population. The differences in clinical presentation underline possible differences in tumor biology. However, differences in outcome may also result of the differences in management, thus the optimal treatment strategy for older adults diagnosed with Ewing sarcoma remains to be defined. Prospective trials are needed to clarify the optimal treatment strategy for patients with EFTs diagnosed after 50 years.

## Methods

### Patients

The study protocol was approved by the relevant regulatory and ethics committee (CCTIRS, CNIL) as well as by the French Sarcoma Group (FSG). In addition, all methods were performed in accordance with the relevant guidelines and regulations. Patients were identified at each participating site using the following criteria: histologically confirmed diagnosis of Ewing sarcoma (by an expert pathologist of the FSG) at age 50 years or older at diagnosis and treated between 2000 and 2012. Seventy-seven patients treated at eight institutions were identified. Data on clinical features, treatment modality and outcomes were extracted from individual patient files. Due to the heterogeneity of chemotherapy regimens, those were classified into two groups: intensive regimens which contained high doses of anthracycline (≥60 mg/m2 per cycle) and high dose alkylating agents (MAI, VIDE, CADO, IVA-IVAD) versus non-intensive which comprised single agent chemotherapy, regimens without anthracycline, as well as “Memphis-like” regimens.

### Statistical analysis

Overall survival (OS) was calculated from the time of diagnosis to date of death or date of last patient contact. Event free survival (EFS) was calculated from date of diagnosis to first event (progression, death) or date of last patient contact or date of death (of any cause). OS- and EFS-rates were estimated according to time using the Kaplan–Meier method. Median follow-up was calculated using a reverse Kaplan–Meier estimate. Only univariate analysis was performed due to the small number of patients in each group. Univariate (Cox proportional hazards regression model) analyses were used to examine the predictive value of significant factors. Associations between different clinico-pathological parameters were estimated by the chi-square test. Statistical analyses were carried out using the SAS software (version 9.4). A *p* value ≤ 0.05 was considered significant.
